# Ensemble learning-assisted prediction of prolonged hospital length of stay after spine correction surgery: a multi-center cohort study

**DOI:** 10.1186/s13018-024-04576-4

**Published:** 2024-02-02

**Authors:** Wenle Li, Yusi Zhang, Xin Zhou, Xubin Quan, Binghao Chen, Xuewen Hou, Qizhong Xu, Weiheng He, Liang Chen, Xiaozhu Liu, Yang Zhang, Tianyu Xiang, Runmin Li, Qiang Liu, Shi-Nan Wu, Kai Wang, Wencai Liu, Jialiang Zheng, Haopeng Luan, Xiaolin Yu, Anfa Chen, Chan Xu, Tongqing Luo, Zhaohui Hu

**Affiliations:** 1 State Key Laboratory of Molecular Vaccinology and Molecular, Diagnostics and Center for Molecular Imaging and Translational Medicine, School of Public Health, Xiamen University, Xiamen, China; 2 Key Laboratory of Neurological Diseases, The Second Affiliated Hospital of Xuzhou Medical University, Xuzhou, Jiangsu China; 3 Department of Spinal Surgery, Guangxi Medical University Affiliated Liuzhou People’s Hospital, Liuzhou, China; 4 Cancer Center, The First Affiliated Hospital of Xi’an Jiaotong University, Xi’an, China; 5 Precision Medicine Center, The First Affiliated Hospital of Xi’an Jiaotong University, Xi’an, China; 6 Department of Medical Oncology, The First Affiliated Hospital of Xi’an Jiaotong University, Xi’an, China; 7 Third Hospital of Shanxi Medical University, Shanxi Bethune Hospital, Shanxi Academy of Medical Sciences, Tongji Shanxi Hospital, Taiyuan, 030032 China; 8 Department of Radiology, The First Dongguan Affiliated Hospital, Guangdong Medical University, Dongguan, China; 9 Department of Radiology, The First Affiliated Hospital of Shenzhen University, Shenzhen Second People’s Hospital, Shenzhen, China; 10 Department of Radiology, People’s Hospital of Ningxia Hui Autonomous Region, Yinchuan, China; 11 Department of Radiology, Hubei Provincial Hospital of Traditional Chinese Medicine, Wuhan, China; 12 Department of Critical Care Medicine, Beijing Shijitan Hospital, Capital Medical University, Beijing, China; 13 Department of Cardiology, The Second Affiliated Hospital of Chongqing Medical University, Chongqing, China; 14 College of Medical Informatics, Chongqing Medical University, Chongqing, China; 15 Medical Data Science Academy, Chongqing Medical University, Chongqing, China; 16 Information Center, The University-Town Hospital of Chongqing Medical University, Chongqing, China; 17 Department of Foot and Ankle Surgery, Honghui Hospital, Xi’an Jiaotong University, Xi’an, Shaanxi Province China; 18 Department of Orthopedics, Xianyang Central Hospital, Xianyang, Shannxi China; 19 Eye Institute of Xiamen University, School of Medicine, Xiamen University, Xiamen, Fujian China; 20 Department of Orthopedics, Shanghai Sixth People’s Hospital Affiliated to Shanghai Jiao Tong University School of Medicine, Shanghai, 200233 China; 21 Cancer Research Center, School of Medicine, Xiamen University, Xiamen, China; 22 Department of Spine Surgery, The Six Affiliated Hospital of Xinjiang Medical University, Urumqi, Xinjiang China; 23 Department of Orthopedics, Affiliated Hospital of Guizhou Medical University, Guiyang, Guizhou China; 24 Department of Orthopedics, Jiangxi Province Hospital of Integrated Chinese and Western Medicine, Nanchang, China

**Keywords:** Spinal deformity, Posterior spinal deformity surgery, Postoperative length of stay, Multimodal, Machine learning

## Abstract

**Purpose:**

This research aimed to develop a machine learning model to predict the potential risk of prolonged length of stay in hospital before operation, which can be used to strengthen patient management.

**Methods:**

Patients who underwent posterior spinal deformity surgery (PSDS) from eleven medical institutions in China between 2015 and 2022 were included. Detailed preoperative patient data, including demographics, medical history, comorbidities, preoperative laboratory results, and surgery details, were collected from their electronic medical records. The cohort was randomly divided into a training dataset and a validation dataset with a ratio of 70:30. Based on Boruta algorithm, nine different machine learning algorithms and a stack ensemble model were trained after hyperparameters tuning visualization and evaluated on the area under the receiver operating characteristic curve (AUROC), precision-recall curve, calibration, and decision curve analysis. Visualization of Shapley Additive exPlanations method finally contributed to explaining model prediction.

**Results:**

Of the 162 included patients, the K Nearest Neighbors algorithm performed the best in the validation group compared with other machine learning models (yielding an AUROC of 0.8191 and PRAUC of 0.6175). The top five contributing variables were the preoperative hemoglobin, height, body mass index, age, and preoperative white blood cells. A web-based calculator was further developed to improve the predictive model's clinical operability.

**Conclusions:**

Our study established and validated a clinical predictive model for prolonged postoperative hospitalization duration in patients who underwent PSDS, which offered valuable prognostic information for preoperative planning and postoperative care for clinicians.

*Trial registration* ClinicalTrials.gov identifier NCT05867732, retrospectively registered May 22, 2023, https://classic.clinicaltrials.gov/ct2/show/NCT05867732.

**Supplementary Information:**

The online version contains supplementary material available at 10.1186/s13018-024-04576-4.

## Introduction

Spinal deformity encompasses a wide range of conditions, such as scoliosis and kyphosis, which is commonly classified as congenital deformity, degenerative deformity, neuromuscular deformity, idiopathic scoliosis, and deformity caused by trauma or syndromes [1, 2]. Adult spinal deformity (ASD) affects 15%–20% of the adult population, with elderly groups observing higher incidence [3]. Given the ASD-related pain, disability, and decreased quality of life, surgical intervention is often necessary to correct these deformities and alleviate associated symptoms [4]. Despite the large patient population involved and the high necessity of surgery, spine correction surgery presents complex challenges to clinicians due to its complicated surgical procedure and relatively high rates of postoperative complications. As a whole, spine correction surgery is still cautiously performed and few related studies have been carried out in clinical practice [5].

In recent years, the concept of Enhanced Recovery After Surgery (ERAS) has been widely popularized in China. Its meticulous management during the perioperative period has great clinical significance and has surprising effects on promoting rapid recovery after surgery. The purpose of ERAS is to shorten the length of stay (LOS) in hospital, save medical costs, and reduce the occurrence of complications and mortality [6]. The postoperative period in hospital plays a crucial role in patient recovery and overall treatment outcomes. Due to the extensive dissection of paraspinal muscles in spinal surgery, patients have a high probability of preoperative and postoperative bleeding and limited postoperative activities. In addition, some patients may be complicated with other chronic diseases before operation, so the risk of prolonged LOS in patients undergoing posterior lumbar interbody fusion surgery is greatly increased. Prolonged LOS in hospital following spinal deformity surgery can lead to increased healthcare costs, patient discomfort, and potential complications [7–10]. Therefore, the ability to accurately predict postoperative hospital stay extension before surgery is paramount in the field of orthopedics, specifically in the management of patients with spinal deformities.

Efforts have been made to identify factors contributing to prolonged LOS in spinal deform patients. Previous studies have reported various predictors, including age, preoperative heart disease, duration of surgery, number of levels fused, and postoperative infection [11–14]. However, the existing literature lacks a comprehensive and validated predictive model that incorporates these predictors to accurately estimate the risk of prolonged LOS in hospital.

In the past, researchers have initially used machine learning (ML) methods to develop prediction models of related factors for patients undergoing correction surgery for spinal deformity, including the risk prediction of prolonged LOS in hospital [15]. However, these models have some limitations, mainly reflected in the single type of ML model, few variables, and the model has not been clinically verified. Ensemble learning [16] is to compare multiple classifiers to complete the learning task together, so as to effectively improve the generalization ability of the single classifier and solve the over-fitting problem. Therefore, in order to further optimize the development of the predictive model for prolonged LOS after posterior spinal deformity surgery (PSDS) in patients with spinal deformities, we integrated a wide range of variables before, during, and after surgery supported by 8 medical institutions. Our goal was to develop a robust and accurate machine learning model that can help clinicians identify high-risk patients with extended hospitalization before the surgical procedure.

## Materials and methods

### Study design and cohorts

We performed a multi-center observational cohort study using clinical record data from the Degenerative Spine Diseases in China (DSDC, NCT05867732) which was contributed by 22 medical institutions from January 2015 to January 2022. It is devoted to evaluating the treatment and prognosis of patients with degenerative spine diseases and constructing an assisted decision-making system. We extracted patients in this project who were diagnosed with spinal deformity and underwent PSDS, totaling 162 patients from January 2015 to January 2022 at eleven hospitals. Our study was approved by the institutional review board of all participating institutions. This study adheres to the Transparent Reporting of a Multivariable Prediction Model for Individual Prognosis or Diagnosis (TRIPOD) reporting guideline [17].

**Fig. 1:**
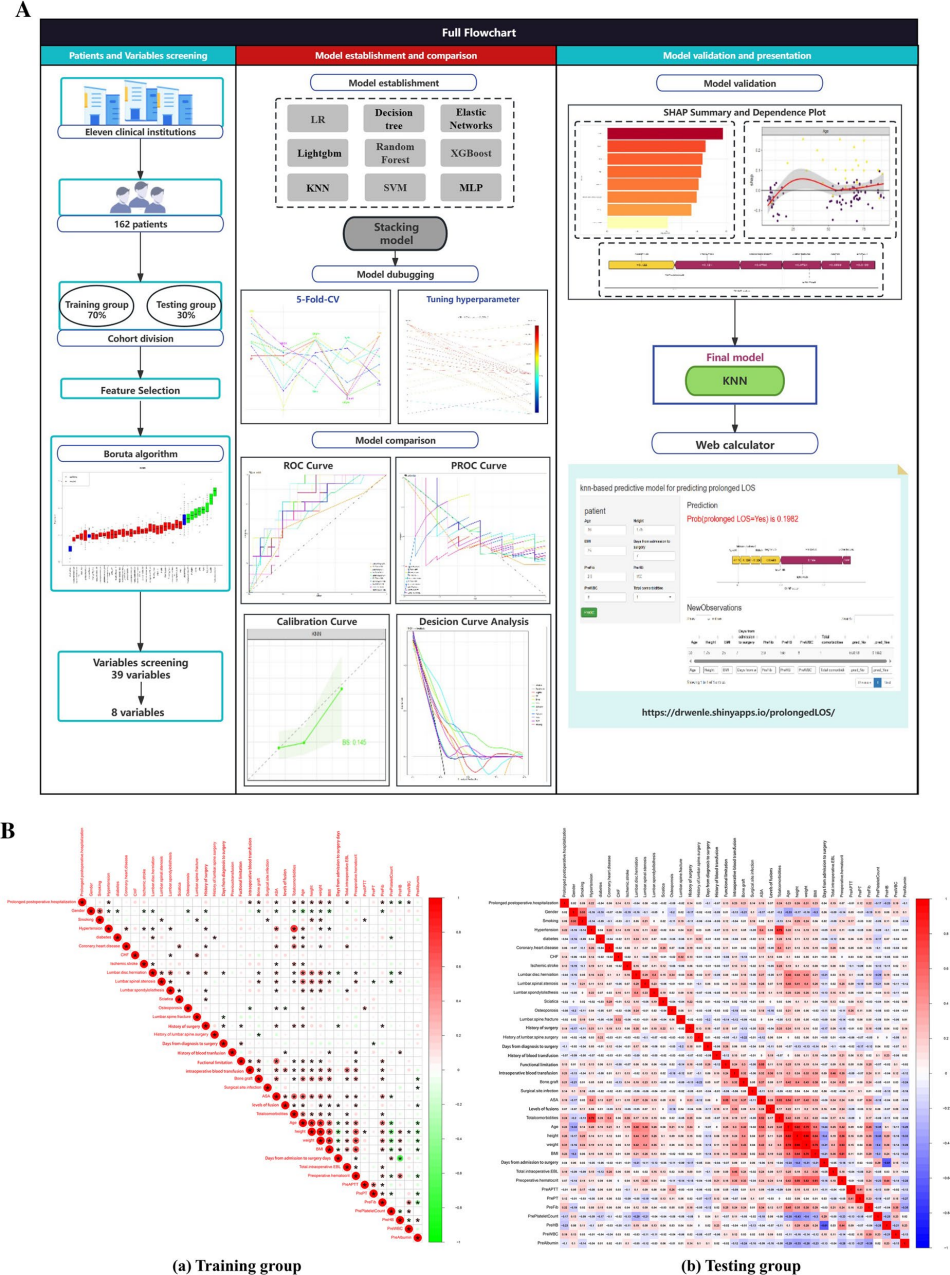
**A** The flowchart of analysis illustration of methodology. LR, logistic regression; KNN, k-Nearest Neighbor; SVM, support vector machine; Lightgbm, Light Gradient boosting machine; XGBoost, eXtreme Gradient Boosting; ROC, receiver operating curve; PROC, precision-recall curve; SHAP, SHapley Additive exPlanations. **B** (a) The correlation between variables in the training group. (b) and in the testing group. Red indicates positive correlation, while blue indicates negative correlation

Inclusion criteria embraced all those patients who underwent posterior spinal fusion surgery for correction of adolescent or adult idiopathic scoliosis, primary adult spine deformity, and post-traumatic spinal deformity in the hospital case system (HIS) system. Exclusion criteria included patients with tumors of spine and missing patient data more than 5%. The study flow is shown in Fig. 1A.

### Outcomes and variables collection

The outcome of interest was prolonged LOS in hospital. A prolonged LOS for posterior spinal surgery was considered greater than or equal to 16 days, meaning that it was higher than the 75th percentage in their LOS distributions. All patients underwent standard posterior spinal surgery for correction and received routine preoperative estimation and intraoperative care.

In total, 38 candidate variables were tested as candidate predictors The general clinical factors included age, gender, height, weight, body mass index (BMI), smoking history, chronic diseases (hypertension, diabetes, coronary heart disease, chronic heart failure, ischemic stroke), other co-morbidities (lumbar disc herniation, lumbar spinal stenosis, lumbar spondylolisthesis, sciatica, osteoporosis, lumbar spine fracture, and total comorbidities), previous surgical history, history of blood transfusion, history of lumbar spine surgery, and preoperative functional limitation. The surgical predictor variable included the blood transfusion, bone graft status, estimated intraoperative blood loss (EBL), the American Society of Anesthesiologists (ASA) score, levels of fusion, surgical site infection, days from diagnosis to surgery, and days from admission to surgery days. And preoperative laboratory factors included hemoglobin (HB), hematocrit, activated partial thromboplastin time (APTT), prothrombin time (PT), fibrinogen (FIB), platelet count, white blood cell (WBC) count, and albumin.

Descriptive statistics were used to characterize the study population at baseline. Qualitative variables were compared using the Pearson Chi-squared test, and normally distributed quantitative variables were compared using t test, while non-normally distributed quantitative data were compared using the Wilcoxon rank test. *P* < 0.05 indicates that all analyses were statistically significant.

These clinical variables were used as the predictor variables for the following model development, and we also used heatmaps to visualize the bivariate relationships between variables (Fig. 1B).

### Model development

#### Model specification

We aimed to identify factors associated with prolonged LOS undergoing PSDS for spinal deformity and establish a robust predictive model for clinicians to identify high-risk patients and intervene at an early stage.

In order to screen the essential variables, we employed the Boruta algorithm, a wrapper algorithm that built around the random forest classification algorithm implemented in the R package Random Forest, to screen independent variables for building a machine learning model to predict the risk of prolonged LOS in hospital [18]. By building shadow features (shadow Max, shadow Mean, shadow Min), Boruta algorithm aims to pick out the relevant features rather than the minimal optimal set of variables. And the algorithm adds extra randomness to the system, making the truly important variables clearer. After multiple random iterations to build multiple random forest classifiers, Boruta's results are generally more stable than those feature selection methods based on a single random forest.

The data were randomly divided into a training dataset and a validation dataset with a ratio of 70:30. In the training set, Bayesian optimization was applied to identify the best parameters for the hyperparameter tuning process of each algorithm and the process was visualized via line charts [19]. Fivefold cross-validation was used to evaluate predictive performance and general error estimates in the model development. Nine kinds of basic ML algorithms were employed to construct the models, namely Logistic Regression [20], Decision Tree [21], Elastic Networks (Enet) [22], K Nearest Neighbors (KNN) [23], Light Gradient boosting machine (Lightgbm) [24], Random Forest [25], eXtreme Gradient Boosting (XGBoost) [26], support vector machines (SVM) [27], Multilayer perceptron (MLP) [28]. Furthermore, based on Lasso regression as meta-model, we establish a stacking ensemble model that can combine information from nine single classifiers mentioned above to predict the patients with prolonged LOS after PSDS.

#### Model validation

We evaluated the predictive performance of our models based on the area under the receiver operating characteristic curve (AUROC) and precision-recall curve (PRAUC), aiming to optimize them for maximum effectiveness. And the AUROC ranges from 0.5 to 1.0, which known as the larger the AUROC accompanied the stronger ability to distinguish. Meanwhile, we utilized decision curve analysis (DCA) to evaluate the clinical utility of multiple ML models, which is a practical tool that calculates the net benefits of predictive models [29].

#### Model presentation

We employed the SHapley Additive exPlanation (SHAP) algorithm to elucidate the prediction model by presenting consistent and locally accurate attribution values (SHAP values) for each feature in each prediction model. These SHAP values assess the significance of the output achieved through the inclusion of a specific feature A, considering all possible combinations of features excluding A [34]. And we randomly selected from the training set and used a SHAP force plot to visualize the examination process of the predictor variables with the outcome of a single patient.

Finally, a web-based calculator based on the prediction model was constructed to enable input of patient preoperative data in order to help clinicians evaluate the risk of postoperative prolonged LOS.

## Results

### Cohort characteristics

In total, 162 patients diagnosed with spine deformity and receiving PSDS were identified in our study. The whole dataset was randomly split into 70% (*n* = 112) and 30% (*n* = 50) in the training group and the validation group, respectively.

Our cohorts were divided into two groups: those who had prolonged LOS in hospital and those who had not (Additional file 1; Table S1). The median age of the total population is 60.6 (IQR = 21.00, 72.00), and the median age of the patients with prolonged LOS and non-prolonged LOS was 59.0 (IQR = 14.00, 71.00) and 68.0 (IQR = 54.00, 75.00), respectively. From the basic personal information, distribution differences were exhibited in the aspects of age, height, weight, and BMI between the two groups. The distribution of comorbidity, like hypertension, differed between the two groups. Among the factors related to the operation, the intraoperative EBL, the proportion of blood transfusion, and bone grafting were different between the two groups. The preoperative Hb and fibrinogen in patients with prolonged LOS were lower than that in patients with normal LOS in hospital. Among the spine-related complications we concerned, there was no significant difference in the distribution of preoperative lumbar disc herniation, dislocation, lumbar fracture, and sciatica between the two groups, and there was no significant difference in the previous history of spinal surgery between the two groups.

### Boruta algorithm screening potential predictor variables

**Fig. 2:**
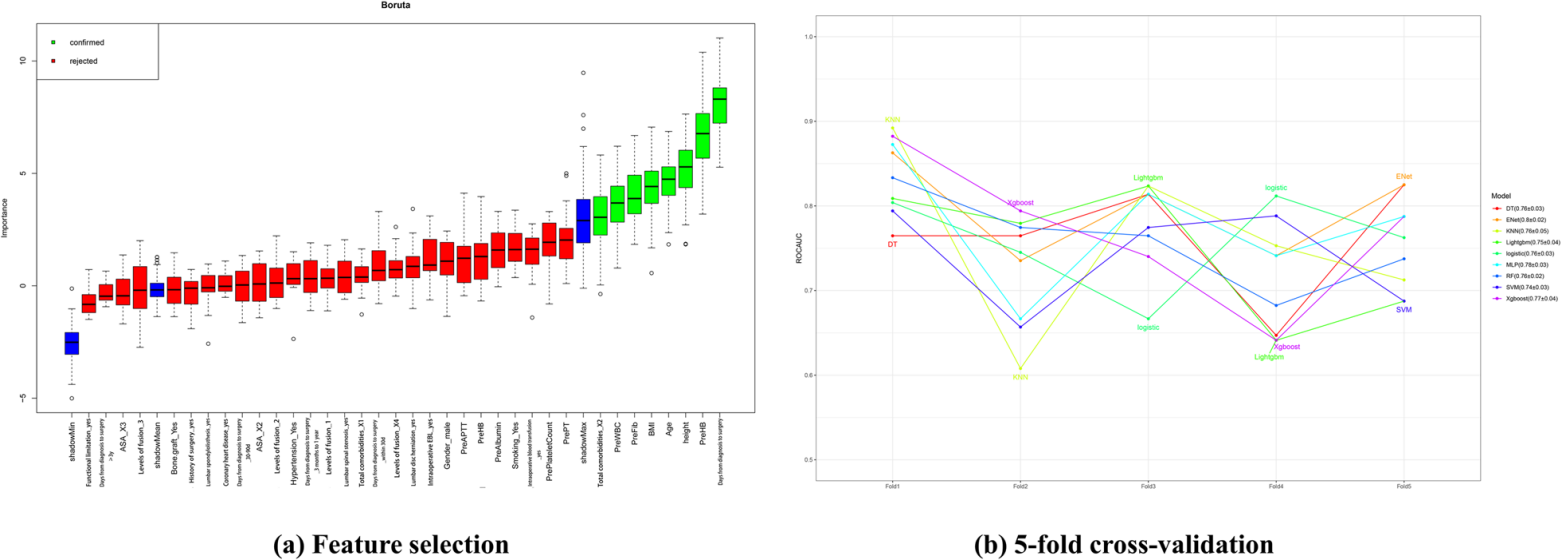
Feature selection and fivefold cross-validation technique. **a** Boruta result plot for training data. Blue boxplots correspond to minimal, average, and maximum Z score of a shadow attribute. Red and green boxplots represent Z scores of, respectively, rejected and confirmed attributes. **b** Accuracy lines plot of nine ML algorithms through fivefold cross-validation

After applying Boruta, eight top relevant features were returned and depicted in increasing order of importance score in Fig. 2a. The eight features were finally used for training and building the proposed ensemble model, including total comorbidities, preoperative WBC, preoperative fibrinogen, BMI, age, height, preoperative HB, and days from admission to surgery.

### Parameter correlation and model performance

**Fig. 3:**
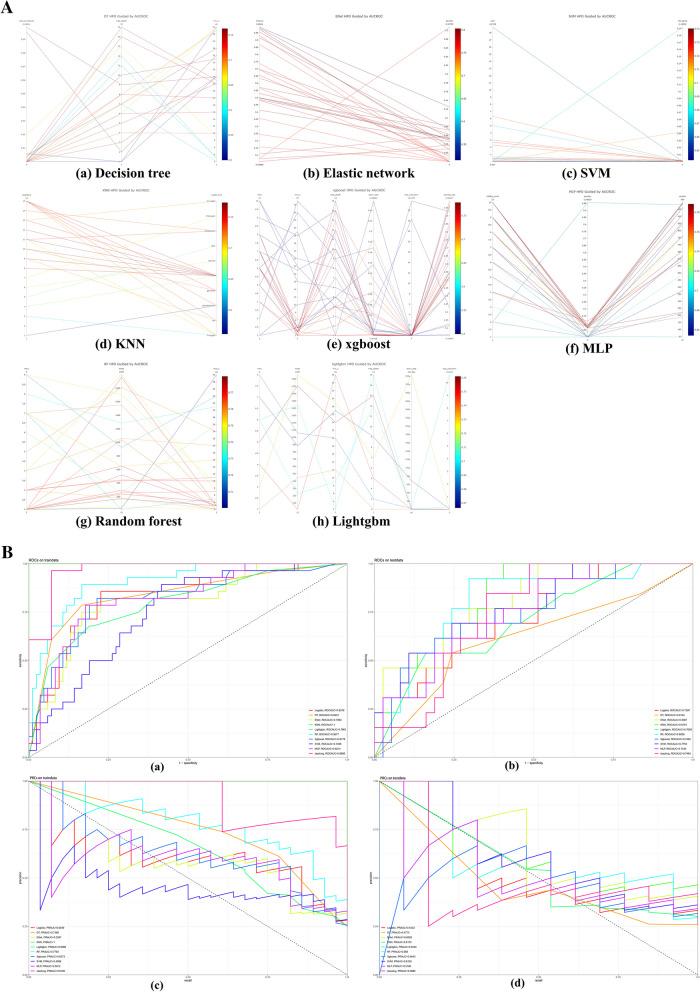

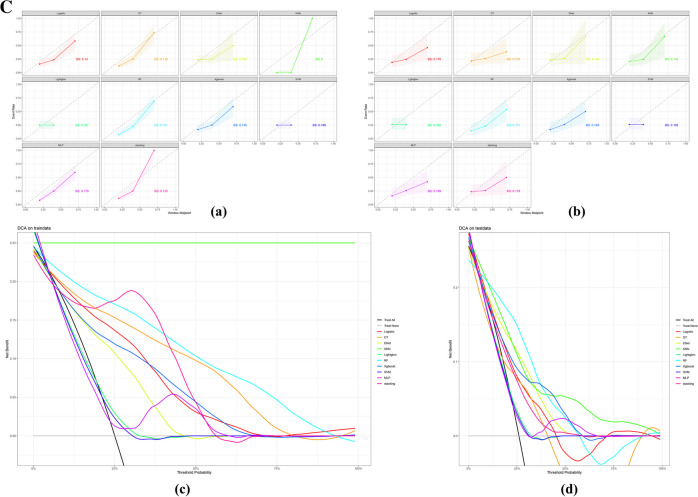
**A** Results of Bayesian hyperparameter optimization. The final hyperparameters were determined by the Optuna hyperparameter tuning framework. The Optuna optimizer maximized the out-of-sample area under the receiver operating characteristic (AUCROC). DT, Decision Tree; Enet, Elastic Networks; KNN, K Nearest Neighbors; Lightgbm, Light Gradient boosting machine; RF, Random Forest; XGBoost, eXtreme Gradient Boosting; SVM, support vector machines; MLP, multilayer perceptron; HPO, hyperparameter optimization. **B** Model performance presentation. (a) The ROC curve of each model in the training group (b) and the testing group; the X-axis represents "1-specificity," while the Y-axis represents sensitivity. (c) The precision-recall of each model in the training group (d) and the testing group, the X-axis represents precision, the Y-axis represents recall. **C** Model performance illustration. (a). The calibration curve of each model in the training group (b) and the testing group (c). The DCA curve of each model in the training group. (d). The DCA curve of each model in the testing group. BS, Best calibration. DCA, Decision curve analysis

To ensure that each machine model achieved the best performance, we further optimized their hyperparameters determined via Bayesian optimization and the visualized process are reported in Fig. 3A. In our research, the most important hyperparameter to tune in regard to objective value was the learning rate. In the training set, fivefold cross-validation was used to evaluate predictive performance and general error estimates in the model development. Figure 2b shows that the Enet model got the highest clinical predictive value, with an AUROC curve of 0.80 ± 0.02, followed by MLP, which had an AUROC curve of 0.78 ± 0.03.

Next, we evaluated machine learning models of prediction that had been trained using 9 different machine learning algorithms and a stack ensemble model. Based on the ROC curves, all ten machine learning algorithms demonstrated excellent predictive performance for postoperative prolonged LOS in both the training and testing sets. Among the training dataset, the KNN model displayed the best performance, followed by the stack model. In terms of the validation dataset, the KNN model continued to exhibit superior predictive performance and model fitting, yielding an AUROC of 0.8191 and PRAUC of 0.6175, followed by the Enet model with an AUROC of 0.8087 and PRAUC of 0.6026. (all ROCs and precision-recall curves see Fig. 3B).

The calibration curve showed a strong correlation between the predicted and actual risks in terms of Brier score (BS), which was used for indicating the calibration ability (Fig. 3Ca-b). The KNN model has the best calibration in the training group (BS = 0) (Fig. 3Ca) and validation group (0.145) (Fig. 3Cb). Then, DCA was used to evaluate the clinical application value of each prediction model. As shown in Fig. 3Cc, d, the X-axis represents the threshold probability of prolonged LOS, and the Y-axis represents the net benefit. KNN model exhibited the continuous maximum benefit in the training group, and KNN, RF, and Xgboost showed greater benefits in different threshold ranges of the test set. However, the benefit of the KNN model was sustained across the full threshold range.

Overall, we can see that the KNN model had the best performance and that there was no overfitting in either the training or validation groups from the comparison of the above models and related parameters. And the model interpretability and development of a web calculator were conducted in accordance with the optimal model (the KNN model) in the following study.

### SHAP analysis of feature importance

**Fig. 4:**
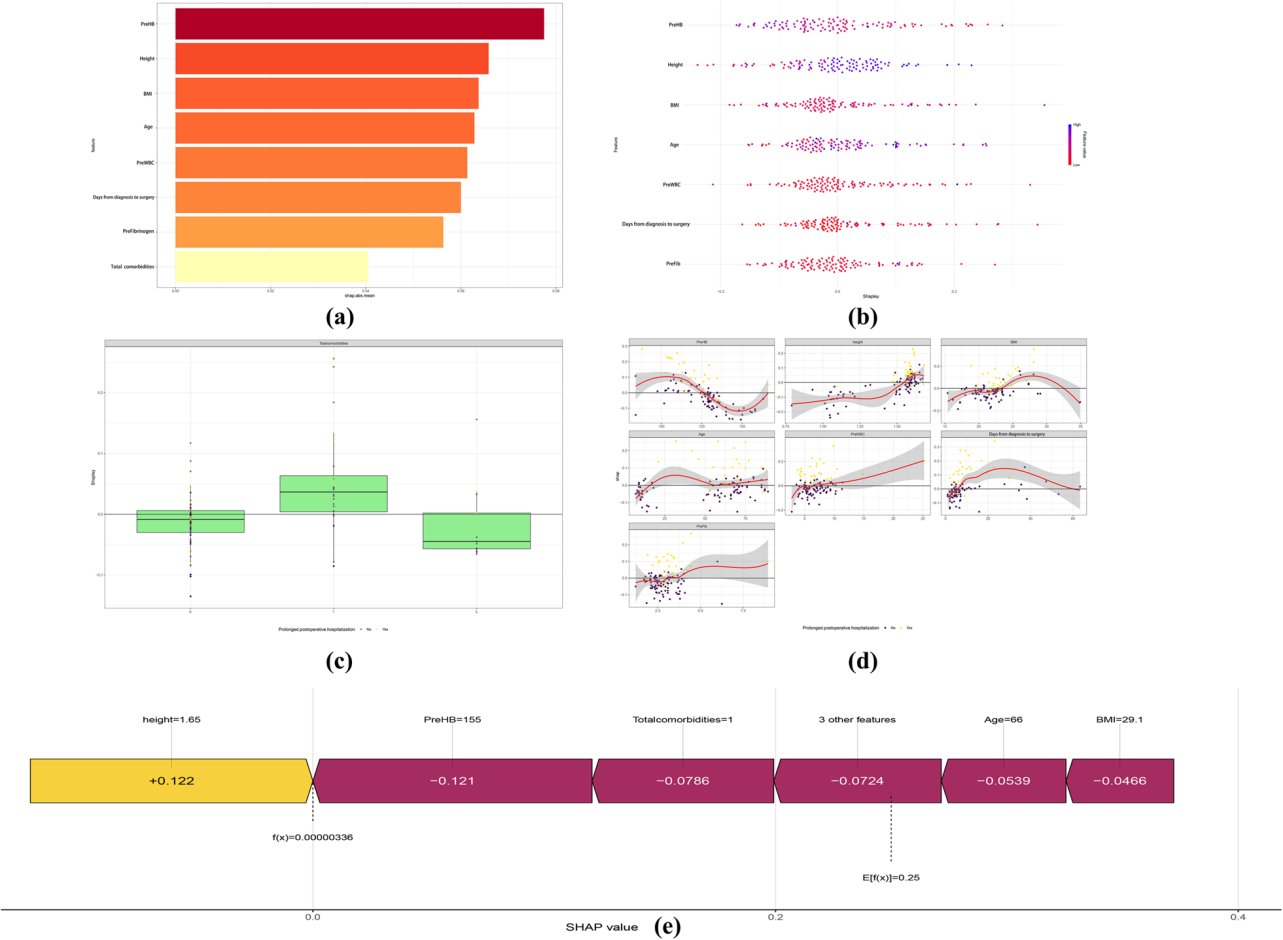
SHAP analysis of feature importance. **a**–**d** Top 8 risk predictors for preoperative prediction of prolonged LOS in hospital after spine deformity correction. **e** The SHAP force plot showed the prediction process of a patient with normal LOS in hospital

SHAP analysis was performed to describe the importance of features in the KNN model according to global feature importance, specific classification results, and individual SHAP force plot, as shown in Fig. 4a–e. Preoperative HB and height were consistently the top two most impactful features. Subsequently, in descending order of importance, BMI, age, preoperative WBC, days from admission to surgery, preoperative fibrinogen, and total comorbidities were identified (Fig. 4a, b).

To further identify the features that have the greatest impact on the predictive model, we depicted a SHAP dependency plot for the eight features of the KNN model (Fig. 4c, d). The plot depicts the individual feature values in relation to the SHAP values in the training dataset, with the y-axis values denoting the SHAP values of individual features and the X-axis denoting the feature values. When the SHAP value exceeds zero, it indicates an increased risk of prolonged LOS in hospital, the higher the SHAP value of the feature, the higher the likelihood of prolonged LOS in hospital.

Figure 4e depicts a SHAP force plot of a patient's hospitalization outcome. The base value *E*[*f*(*X*)] was 0.25, which reflected the average predicted value of the training set. Yellow bars represented the feature's positive contribution to the anticipated value, while red bars represented the feature's negative contribution to the expected value. The 66-year-old patient had high preoperative HB, age, BMI, and total comorbidity scores, with only the height score contributing negatively. The model predicted a lower possibility in the likelihood of a prolonged LOS in hospital based on this information.

### Web-based calculator

**Fig. 5:**
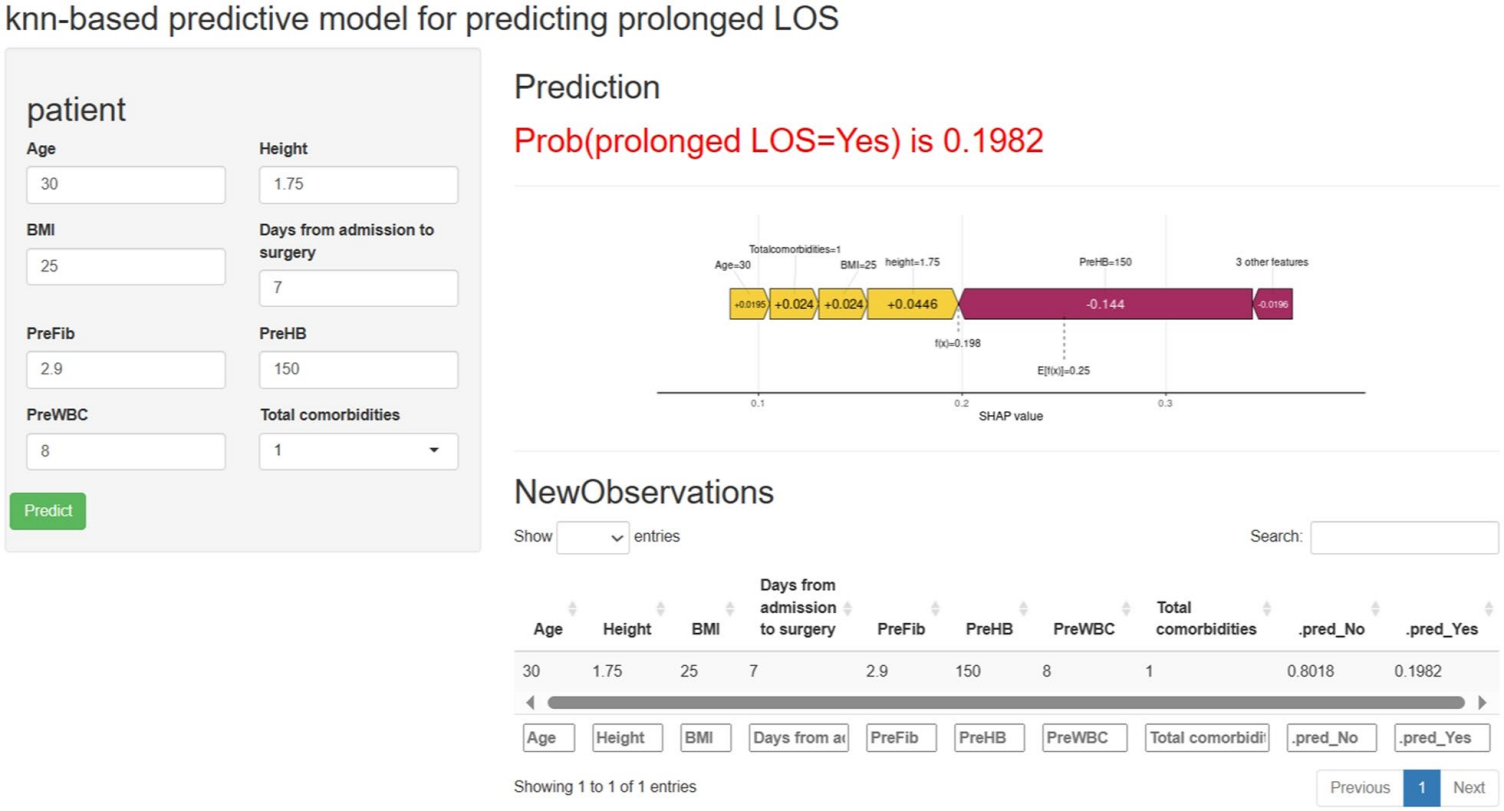
The web calculator of KNN-based predictive model for predicting prolonged LOS and sample exhibition

In addition, we customized a web calculator (https://drwenle.shinyapps.io/prolongedLOS/) based on the features in the KNN model to evaluate the probability of prolonged LOS in hospital. Figure 5 exhibits the interactive interface and an example usage of the web calculator. By inputting the relevant baseline data of the patient to be evaluated, the calculator will visualize the evaluation process in the form of SHAP force plot, and the evaluation history can be subsequently checked in the interface. A machine learning-based predictive model for predicting prolonged length of stay in spine deformity patients underwent spine correction surgery.

## Discussion

By analyzing the data of 162 participants with 38 types of baseline variables, we aimed to establish a robust clinical ML model to predict postoperative prolonged LOS in patients with spinal deformities using ensemble learning algorithms in this multi-center study. Nine metamodels and a stack model were constructed and subjected to hyperparameter optimization based on the Boruta’s feature selection results. Ultimately, the KNN model exhibited the best clinical predictive value in the validation set, with an AUROC and PRAUC of 0.8191 and 0.6175, respectively. A web-based calculator was further developed to assist with the predictive model's clinical operability. Risk evaluation of LOS in hospital is both a challenge and a chance to improve clinical decision-making and care quality, although in-hospital signs remain few. Our application of ensemble learning tried to effectively improve the performance of the single classifier and solve the over-fitting problem. And finally we demonstrated the winner model in accurately predicting postoperative hospital stay duration, providing valuable prognostic information for preoperative planning and postoperative care.

A large number of retrospective and prospective cohort studies have shown that approximately 30% of patients experience perioperative anemia. However, preoperative anemia has not been paid enough attention in clinical management, with the common approach being to correct anemia through blood transfusion or proceed without intervention. This not only increases the risks of intraoperative blood transfusion and surgical complications but also raises the incidence of postoperative organ damage and mortality rates associated with preoperative chronic anemia and intraoperative acute anemia [30]. Studies have shown that preoperational anemia patients have a significantly higher mean LOS (6.5 days; range from 1 to 29 days) than non-anemia patients (4.8 days; range from 1 to 27 days) (*p* ≤ 0.001). Moreover, anemia-induced intraoperative allogeneic blood transfer is also significantly correlated with prolonged LOS [31]. Furthermore, the incidence of preoperative anemia increases with age, with 40% of male patients over 80 years of age undergoing elective cardiac surgery experiencing preoperative anemia [34]. In this predictive model, both factors, anemia and elderly, cumulatively decreasing in the scores led to a higher likelihood to prolonged LOS in hospital. Therefore, clinicians should emphasize the management of preoperative anemia, pay attention to the cause of anemia and correct it in time before operation. Perioperative bleeding and coagulation dysfunction are multifactorial, and in addition to the involvement of hemoglobin, the variables associated with this predictive model also include the lack of preoperative fibrinogen and WBC.

In this study, height entered our feature selection and got a high importance in SHAP value ranking. This phenomenon can be attributed to several factors. Firstly, taller individuals often have longer spinal columns and more severe spinal deformities or degenerative conditions [32, 33], which may require more extensive surgical procedures. The complexity and duration of the surgery itself can contribute to a prolonged recovery period and a subsequent prolonged LOS in hospital. Furthermore, the physiological differences associated with taller stature may contribute to the longer hospital stay. For instance, taller individuals often have a larger body surface area and higher metabolic demands [34]. This increased metabolic rate may lead to a higher risk of postoperative complications, such as infection or respiratory issues, which can prolong the hospital stay for closer monitoring and appropriate treatment. It is important to note that while height has been associated with postoperative LOS in hospital, it is not the sole determinant. Multiple steps and factors contribute to the occurrence of prolonged LOS in spine deformity patients who underwent PSDS. Other patient-specific factors, such as BMI and total comorbidities, should also be considered when assessing the LOS.

In clinical practice, a series of perioperative optimization measures are typically followed according to the ERAS guidelines. However, there are few predictive models available for visualizing postoperative risks in patients, with the venous thromboembolism (VTE) scoring system being the most widely used currently. In this study, we developed a predictive model for clinicians by integrated machine learning algorithms to determine whether the LOS is prolonged in patients who underwent spinal correction surgery. We utilized routine preoperative indicators as variables to visualize the postoperative hospitalization. Although the postoperative prolonged LOS does not directly correspond to treatment decisions, it can enable clinicians to make timely intervention for patients with preoperative indicators that fail to meet the standards. Comparison of multiple models resulted in the final winner model having good sensitivity and accuracy. Given the good calibration scores displayed by the final model, it can also be used to identify patients with similar disease severity for subsequent research purposes. Finally, using the model in conjunction with visualization aids such as SHAP plots could help clinicians identify the specific components that contribute to the severity of the disease. Moving forward, the continued development and refinement of models that use artificial intelligence methodologies have the potential to optimize healthcare resources in the realm of spinal surgery and we believe that hospitals are constructing the infrastructure steadily in order to integrate predictive analytics into HIS systems.

As mentioned above, the prolonged LOS in hospital is a result of multiple factors. Although our research data came from multi-center medical institutions, we mainly collected the preoperative baseline variables of patients recorded by HIS. In the process of postoperative recovery, patients’ psychological and mental state also significantly affects their length of hospitalization. In the subsequent study, our feature collection will further focus on the patient-reported outcome (PRO) in perioperative management and adopt a standard scoring system for variables as far as possible, to improve the accuracy and completeness of the prediction model. Finally, because of the limitations of diagnosis and surgical risks in patients with spine deformity in China, the sample size was necessarily relatively small. However, surgical intervention improves patients’ outcomes and quality of life. So, the sample size may appear relatively small, but we insisted on focusing on this population. It is hoped that our study can draw more researchers’ attention to fully understand risks and make more improvements in this population. Despite its remaining limitations, this study represents the largest, multi-center, retrospective cohort study focused on the prolonged LOS in spine deformity patients who underwent PSDS in China, which could be utilized as a primer for future detailed subgroup study.

## Conclusion

In conclusion, our study established and validated a clinical predictive model for postoperative LOS in hospital extension in patients with spinal deformities using ensemble learning techniques. The comparison of ten machine learning models and the application of the building process together with visualization aids (e.g., for SHAP plot) made our prediction model more comprehensive and robust than previously reported algorithms, which offered valuable prognostic information for preoperative planning and postoperative care for clinicians. Although limitations exist, this research represents a significant step toward improving patient management and resource allocation in the field of spine correction surgery.

### Supplementary Information

**Additional file 1.** Patient demographics and baseline characteristics. 

## Data Availability

The datasets used and/or analyzed during the current study are available from the corresponding author upon reasonable request.

## Abbreviations

ASD: Adult spinal deformity
ERAS: Enhanced Recovery After Surgery
LOS: Length of stay
ML: Machine learning
PSDS: Posterior spinal deformity surgery
TRIPOD: Transparent Reporting of a Multivariable Prediction Model for Individual Prognosis or Diagnosis
HIS: Hospital case system
BMI: Body mass index
EBL: Estimated intraoperative blood loss
ASA: American Society of Anesthesiologists
HB: Hemoglobin
APTT: Activated partial thromboplastin time
PT: Prothrombin time
FIB: Fibrinogen
WBC: White blood cell
Enet: Elastic Networks
KNN: K Nearest Neighbors
Lightgbm: Light Gradient boosting machine
XGBoost: EXtreme Gradient Boosting
SVM: Support vector machines
MLP: Multilayer perceptron
ROC: Receiver operating characteristic curve
AUROC: Area under the receiver operating characteristic curve
PRAUC: Area under the precision-recall curve
DCA: Decision curve analysis
SHAP: SHapley Additive exPlanation
BS: Brier score
