# Supplementary material for: Ensemble learning-assisted prediction of prolonged hospital length of stay after spine correction surgery: a multi-center cohort study
Source: J Orthop Surg Res. 2024 Feb 2;19:112. doi: 10.1186/s13018-024-04576-4 (PMC10838003; doi:10.1186/s13018-024-04576-4)
Supplement: Supplementary file 1 — Additional file 1. Patient demographics and baseline characteristics. [file 13018_2024_4576_MOESM1_ESM.docx]

**Table 1.** Patient demographics and baseline characteristics

| **Characteristic** | **Prolonged length of stay in hospital** | | | **p-value** |
| --- | --- | --- | --- | --- |
|  | **Overall (N = 162^1^)** | **No (N = 121^1^)** | **Yes (N = 41^1^)** |  |
| **Gender** |  |  |  |  |
| Male | 65 (40%) | 48 (40%) | 17 (41%) | 0.839 |
| Female | 97 (60%) | 73 (60%) | 24 (59%) |  |
| **Smoking** |  |  |  |  |
| No | 136 (84%) | 104 (86%) | 32 (78%) | 0.234 |
| Yes | 26 (16%) | 17 (14%) | 9 (22%) |  |
| **Hypertension** |  |  |  |  |
| No | 121 (75%) | 97 (80%) | 24 (59%) | 0.006 |
| Yes | 41 (25%) | 24 (20%) | 17 (41%) |  |
| **Diabetes** |  |  |  |  |
| No | 156 (96%) | 116 (96%) | 40 (98%) | ＞0.999 |
| Yes | 6 (4%) | 5 (4%) | 1 (2%) |  |
| **Coronary heart disease** |  |  |  |  |
| No | 154 (95%) | 116 (96%) | 38 (93%) | 0.418 |
| Yes | 8 (5%) | 5 (4%) | 3 (7%) |  |
| **CHF^2^** |  |  |  |  |
| No | 161 (99%) | 121 (100%) | 40 (98%) | 0.253 |
| Yes | 1 (1%) | 0 (0%) | 1 (2%) |  |
| **Ischemic stroke** |  |  |  |  |
| No | 154 (95%) | 117 (97%) | 37 (90%) | 0.113 |
| Yes | 8 (5%) | 4 (3%) | 4 (10%) |  |
| **Lumbar disc herniation** |  |  |  |  |
| No | 97 (60%) | 71 (59%) | 26 (63%) | 0.593 |
| Yes | 65 (40%) | 50 (41%) | 15 (37%) |  |
| **Lumbar spinal stenosis** |  |  |  |  |
| No | 105 (65%) | 81 (67%) | 24 (59%) | 0.330 |
| Yes | 57 (35%) | 40 (33%) | 17 (41%) |  |
| **Lumbar spondylolisthesis** |  |  |  |  |
| No | 131 (81%) | 97 (80%) | 34 (83%) | 0.698 |
| Yes | 31 (19%) | 24 (20%) | 7 (17%) |  |
| **Sciatica** |  |  |  |  |
| No | 157 (97%) | 117 (97%) | 40 (98%) | ＞0.999 |
| Yes | 5 (3%) | 4 (3%) | 1 (2%) |  |
| **Osteoporosis** |  |  |  |  |
| No | 153 (94%) | 113 (93%) | 40 (98%) | 0.451 |
| Yes | 9 (6%) | 8 (7%) | 1 (2%) |  |
| **Lumbar spine fracture** |  |  |  |  |
| No | 153 (94%) | 114 (94%) | 39 (95%) | ＞0.999 |
| Yes | 9 (6%) | 7 (6%) | 2 (5%) |  |
| **Surgical history** |  |  |  |  |
| No | 120 (74%) | 94 (78%) | 26 (63%) | 0.072 |
| Yes | 42 (26%) | 27 (22%) | 15 (37%) |  |
| **History of lumbar spine surgery** |  |  |  |  |
| No | 159 (98%) | 119 (98%) | 40 (98%) | ＞0.999 |
| Yes | 3 (2%) | 2 (2%) | 1 (2%) |  |
| **History of blood transfusion** |  |  |  |  |
| No | 160 (99%) | 119 (98%) | 41 (100%) | ＞0.999 |
| Yes | 2 (1%) | 2 (2%) | 0 (0%) |  |
| **Preoperative functional limitation** |  |  |  |  |
| No | 78 (48%) | 63 (52%) | 15 (37%) | 0.086 |
| Yes | 84 (52%) | 58 (48%) | 26 (63%) |  |
| **Intraoperative transfusion** |  |  |  |  |
| No | 105 (65%) | 86 (71%) | 19 (46%) | 0.004 |
| Yes | 57 (35%) | 35 (29%) | 22 (54%) |  |
| **Intraoperative bone grafting** |  |  |  |  |
| No | 46 (28%) | 41 (34%) | 5 (12%) | 0.008 |
| Yes | 116 (72%) | 80 (66%) | 36 (88%) |  |
| **Surgical site infection** |  |  |  |  |
| No | 161 (99%) | 121 (100%) | 40 (98%) | 0.253 |
| Yes | 1 (1%) | 0 (0%) | 1 (2%) |  |
| **ASA^3^** |  |  |  |  |
| 1 | 41 (25%) | 36 (30%) | 5 (12%) | 0.062 |
| 2 | 83 (51%) | 61 (50%) | 22 (54%) |  |
| 3 | 35 (22%) | 22 (18%) | 13 (32%) |  |
| 4 | 3 (2%) | 2 (2%) | 1 (2%) |  |
| **Levels of fusion** |  |  |  |  |
| 0 | 35 (22%) | 25 (21%) | 10 (24%) | 0.212 |
| 1 | 38 (23%) | 30 (25%) | 8 (20%) |  |
| 2 | 39 (24%) | 32 (26%) | 7 (17%) |  |
| 3 | 27 (17%) | 21 (17%) | 6 (15%) |  |
| ≥4 | 23 (14%) | 13 (11%) | 10 (24%) |  |
| **Total comorbidities** |  |  |  |  |
| 0 | 106 (65%) | 85 (70%) | 21 (51%) | 0.003 |
| 1 | 34 (21%) | 26 (21%) | 8 (20%) |  |
| ≥2 | 22 (14%) | 10 (8%) | 12 (29%) |  |
| **Days from diagnosis to surgery** |  |  |  | 0.353 |
| Within 30 days | 39 (24%) | 28 (23%) | 11 (27%) |  |
| 30 to 90 days | 35 (22%) | 24 (20%) | 11 (27%) |  |
| 3 months to 1 year | 22 (14%) | 15 (12%) | 7 (17%) |  |
| 1 to 3 years | 33 (20%) | 29 (24%) | 4 (10%) |  |
| More than 3 years | 33 (20%) | 25 (21%) | 8 (20%) |  |
| **Age** |  |  |  |  |
| Median (IQR) | 60.6 (21.00, 72.00) | 59.0 (14.00, 71.00) | 68.0 (54.00, 75.00) | 0.009 |
| **Height** |  |  |  |  |
| Median (IQR) | 1.6 (1.48, 1.60) | 1.6 (1.23, 1.60) | 1.6 (1.56, 1.60) | 0.044 |
| **Weight** |  |  |  |  |
| Median (IQR) | 56.3 (44.75, 60.97) | 55.5 (25.79, 59.16) | 59.8 (54.83, 63.49) | ＜0.001 |
| **BMI^4^** |  |  |  |  |
| Median (IQR) | 22.2 (20.73, 23.46) | 21.8 (20.23, 22.97) | 23.2 (22.14, 24.84) | ＜0.001 |
| **Days from admission to surgery** |  |  |  |  |
| Median (IQR) | 5.1 (4.00, 8.01) | 5.0 (3.21, 7.00) | 7.9 (5.48, 11.74) | ＜0.001 |
| **Intraoperative EBL^5^** |  |  |  |  |
| Median (IQR) | 421.7 (283.71, 646.49) | 400.0 (265.40, 567.05) | 600.0 (300.00, 1,000.00) | 0.004 |
| **Preoperative hematocrit** |  |  |  |  |
| Median (IQR) | 23.7 (18.88, 36.59) | 22.7 (17.54, 37.20) | 26.0 (20.69, 33.10) | 0.131 |
| **Pre APTT^6^** |  |  |  |  |
| Mean (SD) | 29.8 (3.75) | 29.8 (3.61) | 29.8 (4.20) | 0.946 |
| **Pre PT^7^** |  |  |  |  |
| Median (IQR) | 11.4 (10.80, 11.98) | 11.4 (10.90, 11.81) | 11.5 (10.70, 12.70) | 0.459 |
| **Preoperative Fibrinogen** |  |  |  |  |
| Median (IQR) | 2.8 (2.39, 3.27) | 2.7 (2.36, 3.13) | 3.1 (2.80, 3.58) | 0.004 |
| **Preoperative Platelet Count** |  |  |  |  |
| Median (IQR) | 223.5 (180.25, 261.00) | 226.0 (176.00, 274.00) | 214.0 (183.00, 235.00) | 0.097 |
| **Preoperative** **Hemoglobin** |  |  |  |  |
| Median (IQR) | 128.7 (114.14, 134.24) | 129.5 (121.00, 134.83) | 118.8 (109.59, 129.34) | 0.001 |
| **Preoperative WBC^8^** |  |  |  |  |
| Median (IQR) | 6.6 (5.08, 7.77) | 6.5 (5.15, 7.68) | 6.7 (4.96, 8.80) | 0.370 |
| **Preoperative Albumin** |  |  |  |  |
| Median (IQR) | 41.3 (38.83, 43.64) | 41.5 (38.50, 44.17) | 40.6 (39.26, 41.71) | 0.150 |

Abbreviation: ^1^n (%); ^2^CHF: Chronic heart failure; ^3^ASA: American society of Anesthesiologists physical status classification system; ^4^BMI: Body mass index; ^5^EBL: estimated blood loss; ^6^APTT: Activated partial thromboplastin time; ^7^PT: Prothrombin time; ^8^WBC: White blood cell.
